# Associations of maternal gestational hypertension with high blood pressure and overweight/obesity in their adolescent offspring: a retrospective cohort study

**DOI:** 10.1038/s41598-022-07903-z

**Published:** 2022-03-08

**Authors:** Renata Kuciene, Virginija Dulskiene

**Affiliations:** Institute of Cardiology, Medical Academy, Lithuanian University of Health Sciences, Sukileliu 15, 50162 Kaunas, Lithuania

**Keywords:** Cardiology, Risk factors

## Abstract

Maternal hypertensive disorders during pregnancy may have an impact on fetal development and the health of the offspring in later life. The aim of the study was to evaluate the associations of maternal gestational hypertension (GH) with high blood pressure (HBP) (prehypertension/hypertension) and overweight/obesity in their adolescent offspring at the age of 12 to 15 years. We analyzed data of 4819 participants born in Kaunas city during 1995–1998 who were included in the study “Prevalence and Risk Factors of HBP in 12–15-Year-Old Lithuanian Children and Adolescents”. The diagnosis of maternal gestational hypertension was obtained from medical records. Associations of maternal GH with their offspring’s HBP and overweight/obesity in adolescence were assessed by multivariate logistic regression analysis. Among 4819 adolescents of 12–15 years of age, 25.7% had HBP, 12% had overweight, and 2.5% had obesity. Of 4819 mothers, 92.3% were normotensive during pregnancy, and 7.7% had GH. In the multivariate analysis after adjustment for age, sex, birth weight, adolescent BMI, and maternal pre/early pregnancy BMI, adolescent offspring born to mothers with GH had higher odds of prehypertension, hypertension, and prehypertension/hypertension (aOR 1.58; 95% CI 1.13–2.22; aOR 1.87; 95% CI 1.41–2.47; and aOR 1.76, 95% CI 1.39–2.24; respectively), compared to the offspring of normotensive mothers. After adjustment for age, sex, birth weight, and maternal pre/early pregnancy BMI, a significant association was found between maternal GH and the offspring’s overweight/obesity in adolescence (aOR 1.41; 95% CI 1.04–1.91). The findings of this study suggest that maternal GH is associated with an increased odds of HBP (prehypertension and hypertension, both separately and combined) and overweight/obesity in their offspring during adolescence.

## Introduction

Hypertension can be caused by various individual and environmental risk factors or their interactions, including genetics, behavioral risk factors, unhealthy diet, tobacco use, harmful use of alcohol, physical inactivity, unhealthy environments, and exposure to persistent stress^1^. High blood pressure (HBP) increases the risk of ischemic heart disease, stroke, rheumatic heart disease, renal diseases, and chronic kidney disease^2,3^, and is one of the leading risk factors for global mortality, accounting for 9.4 million deaths and 7% of global disability-adjusted life-years in 2010^4^. HBP in adolescence is significantly associated with left ventricular hypertrophy, increased carotid intima-media thickness, high pulse wave velocity, and it can predict incident cardiovascular disease and all-cause mortality in adulthood^5^. Hypertension among children and adolescents is increasing worldwide and is becoming a significant and rising health problem^6–8^. A comprehensive systematic review of fifty-five studies with a total of 122053 adolescents found that the prevalence of HBP was 11.2%^9^.

Worldwide, obesity and overweight remain among the most challenging and important problems of public health among children, adolescents^10–12^, and adults^11^. The analysis conducted by the NCD Risk Factor Collaboration (NCD-RisC) showed that from 1975 to 2016, the prevalence of obesity increased worldwide among children and adolescents aged 5–19 years: from 0.9% (0.5–1.3) to 7.8% (6.7–9.1) in boys, and from 0.7% (0.4–1.2) to 5.6% (4.8–6.5) in girls^11^. Obesity is known to be a multifactorial disease caused by a complex interaction between genetics, lifestyle, and environmental factors^13,14^. Children with overweight or obesity are likely to retain these problems in adulthood, which may result in various health issues^15^. Obesity is associated with an increased risk for cardiovascular disease, obesity-associated cardiomyopathy, essential hypertension, coronary artery disease, left ventricular hypertrophy, stroke, cancer, gastrointestinal disorders, type 2 diabetes mellitus, prediabetes, metabolic syndrome, adverse obstetric and perinatal outcomes, obstructive sleep apnea, reproductive disorders, and psychological problems^16^. Moreover, overweight and obesity are related to increased all-cause mortality worldwide^17^.

Hypertensive disorders during pregnancy, including chronic (pre-existing) hypertension, gestational hypertension (GH) (de novo hypertension after 20 weeks of gestation, and then the blood pressure normalizes post-partum), preeclampsia-eclampsia and chronic hypertension with superimposed preeclampsia-eclampsia^18^ affect about 5–10% of pregnancies^19^. According to the WHO systematic review and analysis, between 2003 and 2009, hypertensive disorders during pregnancy were the second direct cause of maternal death worldwide, accounting for 14% of maternal deaths^20^. In addition, they contributed to fetal and neonatal morbidity and mortality^21^. Gestational hypertension is one of the most common hypertensive disorders during pregnancy^22–24^ and has been linked to adverse health outcomes in mothers^23,25^ and their offspring in later life^26^. Women with GH have a higher risk of developing hypertension^27^, cardiovascular disease^28,29^, ischemic heart disease, myocardial infarction, myocardial infarction-related death, heart failure, ischemic cerebrovascular disease, chronic kidney disease, and diabetes mellitus in later life^25^. A systematic review of the literature and a meta-analysis demonstrated that maternal GH was associated with an increased risk of cardiovascular diseases (all stroke subtypes, thrombotic stroke, and hypertension)^30^ and type 2 diabetes^31^ in the adult offspring. Children born to gestational hypertensive mothers are more prone to HBP^32^, obesity^33^, and impairments in cognitive development^34^.

Epidemiological studies have investigated the associations of maternal GH with HBP among children and adolescents^32,35–42^. However, evidence on the offspring’s risk of prehypertension and hypertension analyzed separately is lacking. Little is known about the link between maternal GH and the offspring’s overweight/obesity during adolescence. These associations between the above-mentioned risk factors among children and adolescents aged 12–15 years have not been studied in Lithuania before. Hence, early detection of the risk factors associated with HBP and overweight/obesity as well as an early identification of children at an increased cardiometabolic risk is essential for the prevention of metabolic and cardiovascular diseases in later life and can reduce the burden of these diseases for individuals, for society, and for healthcare systems.

The aim of this study was to evaluate the associations of maternal GH with HBP and overweight/obesity in their offspring at the age of 12 to 15 years. We hypothesized that maternal GH could be positively associated with higher odds of their adolescent offspring having prehypertension and/or hypertension and overweight/obesity, compared to those born to normotensive mothers.

## Results

Among the 4819 women, 4448 (92.3%) had normotension during pregnancy, and 371 (7.7%) had GH. Table 1 shows the distributions of characteristics of mothers during pregnancy and of their offspring at birth and in adolescence by whether the mother had normotension or GH during pregnancy. The mean age at delivery of mothers with GH was significantly greater compared to that in the group without GH. Women aged 35 years or older were more likely to have GH compared to women who were 20–34 years of age. Mothers with GH had a higher prevalence of heart diseases and obesity during pre- and early-pregnancy than women without GH did. There were no significant differences in the prevalence of diabetes mellitus between the groups. The mean values of gestational age and Apgar scores at 1 min and 5 min were significantly lower in the offspring of mothers with GH compared to the offspring of mothers without GH. A high BP (prehypertension/hypertension) and a high BMI status (overweight/obesity) were more prevalent among adolescents whose mothers had GH than among adolescents whose mothers were normotensive. The adolescents of mothers with GH had significantly higher mean BP (SBP, DBP, MAP, and PP), BMI, TMI, WC, and WHtR, compared to the reference group.

**Table 1:** Maternal and offspring characteristics according to maternal normotension or gestational hypertension during pregnancy.

Variables	Normotensive (n = 4448)	GH (n = 371)	p*
**Maternal characteristics**			
Maternal age at delivery (years), n (%)			
< 20	346 (7.8)	19 (5.1)	0.003
20–34	3742 (84.1)	305 (82.2)	
≥ 35	360 (8.1)	47 (12.7)*	
Maternal age at delivery (years), mean ± SD	26.81 ± 5.25	27.54 ± 5.62	0.033
Maternal education			
Primary/secondary	2325 (52.5)	187 (50.4)	0.441
Advanced vocational/higher	2105 (47.5)	184 (49.6)	
Maternal diabetes mellitus			
No	4410 (99.1)	366 (98.7)	0.332
Yes	38 (0.9)	5 (1.3)	
Maternal body weight status, n (%)			
No obesity	4154 (93.4)	287 (77.4)*	< 0.001
Obesity	294 (6.6)	84 (22.6)*	
Maternal heart diseases, n (%)			
No	4001 (90.0)	319 (86.0)*	0.016
Yes	447 (10.0)	52 (14.0)*	
**Neonates’ characteristics**			
Sex, n (%)			
Boys	2042 (45.9)	165 (44.5)	0.594
Girls	2406 (54.1)	206 (55.5)	
Birth weight categories (g), n (%)			
≤ 4000	3856 (86.7)	322 (86.8)	0.956
> 4000	592 (13.3)	49 (13.2)	
Birth weight for gestational age			
SGA	378 (8.5)	40 (10.8)	0.320
AGA	3602 (81.0)	292 (78.7)	
LGA	468 (10.5)	39 (10.5)	
Birth weight (g), mean ± SD	3498.77 ± 506.77	3458.08 ± 559.36	0.182
Gestational age (weeks), mean ± SD	39.50 ± 1.53	39.36 ± 1.47	0.016
Birth length (cm), mean ± SD	50.97 ± 2.31	50.95 ± 2.18	0.928
Weight/length ratio, (kg/m), mean ± SD	6.84 ± 0.82	6.76 ± 0.92	0.130
BMI, (kg/m^2^), mean ± SD	13.41 ± 1.40	13.25 ± 1.54	0.084
PI, (g/cm^3^), mean ± SD	2.63 ± 0.27	2.60 ± 0.28	0.055
Apgar scores, mean (SD)			
1-min Apgar score, mean ± SD	8.43 ± 1.08	8.25 ± 1.15	0.002
5-min Apgar score, mean ± SD	9.11 ± 0.96	9.01 ± 0.79	0.001
**Adolescents’ characteristics**			
Age (years), n (%)			
12–13	2060 (46.3)	176 (47.4)	0.676
14–15	2388 (53.7)	195 (52.6)	
BP categories, n (%)			
NBP	3347 (75.2)	234 (63.1)*	< 0.001
Prehypertension	444 (10.0)	49 (13.2)*	
Hypertension	657 (14.8)	88 (23.7)*	
BP categories, n (%)			
NBP	3347 (75.2)	234 (63.1)*	< 0.001
HBP	1101 (24.8)	137 (36.9)*	
BMI categories, n (%)			
Normal weight	3824 (86.0)	298 (80.3)*	0.003
Overweight/obesity	624 (14.0)	73 (19.7)*	
Age (years), mean ± SD	13.61 ± 1.04	13.55 ± 1.06	0.314
Weight (kg), mean ± SD	54.34 ± 14.65	55.57 ± 12.60	0.051
Height (cm), mean ± SD	165.64 ± 9.58	165.56 ± 9.33	0.912
BMI (kg/m^2^), mean ± SD	19.67 ± 5.51	20.12 ± 3.58	0.014
TMI (kg/m^3^), mean ± SD	11.90 ± 3.78	12.16 ± 2.15	0.020
WC (cm), mean ± SD	67.15 ± 7.65	68.27 ± 8.35	0.015
WHtR, mean ± SD	0.406 ± 0.04	0.413 ± 0.05	0.009
SBP (mm Hg), mean ± SD	118.71 ± 14.51	122.86 ± 15.48	< 0.001
DBP (mm Hg), mean ± SD	65.86 ± 7.77	67.36 ± 7.99	0.001
MAP (mm Hg), mean ± SD	83.48 ± 8.83	85.86 ± 8.99	< 0.001
PP (mm Hg), mean ± SD	52.25 ± 11.69	55.05 ± 13.57	< 0.001

*GH* gestational hypertension, *SGA* small for gestational age, *AGA* appropriate for gestational age, *LGA* large for gestational age, *BMI* body mass index, *PI* ponderal index, *NBP* normal blood pressure, *HBP* high blood pressure, *WC* waist circumference, *TMI* tri-ponderal mass index, *WHtR* waist-to-height ratio, *SBP* systolic blood pressure, *DBP* diastolic blood pressure, *MAP* mean arterial pressure, *PP* pulse pressure.

Values are numbers (percentages) and mean ± SD (standard deviation).

*P < 0.05 vs. the normotensive group (z test).

The prevalence of HBP among adolescents aged 12–15 years was 25.7% (prehypertension, 10.2%; hypertension, 15.5%). Compared to the normotensive group, participants with HBP demonstrated significantly higher mean values of birth weight, birth length, weight/length ratio, and adolescent age, weight, height, BMI, TMI, WC, WHtR, and BP measurements (Supplementary Table 1). Mean gestational age was significantly lower in subjects with HBP than in subjects with NBP. Offspring in the HBP group had a significantly higher rate of maternal GH, maternal diabetes mellitus, maternal pre/early pregnancy obesity, higher birth weight (> 4000 g) and LGA, and adolescent overweight/obesity, compared to the normotensive group. Boys were more often hypertensive than girls were. In addition, participants aged 14–15 years were significantly more likely to have HBP than younger participants aged 12–13 years.

The study showed that 12.0% of the adolescents had overweight, and 2.5% had obesity. Mean values of birth weight, birth length, weight/length ratio, PI, BMI at birth and in adolescence, weight, height, TMI, WC, WHtR, and all BP parameters were significantly higher in subjects with overweight/obesity than in the normal weight group (Supplementary Table 2). The proportion of adolescents who had overweight/obesity was higher among mothers with GH and pre/early pregnancy obesity, in the group of birth weight more than 4000 g and the LGA groups, in the HBP group, and in male offspring, in comparison with their normal weight counterparts. Overweight/obesity was more common among younger adolescents than among the older ones.

Tables 2 and 3 present crude and adjusted logistic regression analyses of the associations of maternal GH with offspring HBP and overweigh/obesity during adolescence. Univariate analysis showed that maternal GH was significantly associated with HBP (prehypertension: OR 1.58, hypertension: OR 1.92, and prehypertension/hypertension: OR 1.78) (Table 2) and overweight/obesity (OR 1.50) (Table 3) in adolescents. Adjustments for potential confounding factors (age and sex) in the first models did not change the significance of the associations, and adjusted odd ratios slightly increased for HBP and for overweight/obesity. According to the final multivariate models, after adjustment for age, sex, birth weight, adolescent BMI, and maternal pre/early pregnancy BMI, offspring of mothers with GH had significantly higher odds of prehypertension, hypertension, and prehypertension/hypertension (aOR 1.58; aOR 1.87; and aOR 1.76, respectively) (Table 2). After adjusting for age, sex, birth weight, and maternal pre/early pregnancy BMI, the adjusted odds ratio decreased to 1.41 (Table 3), but the association of maternal GH with overweight/obesity of their adolescent offspring remained significant.

**Table 2:** Associations of maternal gestational hypertension with HBP in adolescent offspring (univariate and multivariate analyses).

Variables	Prehypertension			Hypertension			Prehypertension/Hypertension		
	OR (95% CI)	aOR^1^ (95% CI)	aOR^2^ (95% CI)	OR (95% CI)	aOR^1^ (95% CI)	aOR^2^ (95% CI)	OR (95% CI)	aOR^1^ (95% CI)	aOR^2^ (95% CI)
Gestational hypertension									
No	1.00	1.00	1.00	1.00	1.00	1.00	1.00	1.00	1.00
Yes	1.58 (1.14–2.18)	1.62 (1.17–2.25)	1.58 (1.13–2.22)	1.92 (1.48–2.48)	2.02 (1.55–2.64)	1.87 (1.41–2.47)	1.78 (1.43–2.22)	1.86 (1.48–2.34)	1.76 (1.39–2.24)
	P = 0.006	P = 0.004	P = 0.008	P < 0.001	P < 0.001	P < 0.001	P < 0.001	P < 0.001	P < 0.001

*OR* crude odds ratio, *aOR*^*1*^ adjusted odds ratios for age and sex, *aOR*^*2*^ adjusted odds ratios for age, sex, birth weight, adolescent BMI, and maternal pre/early pregnancy BMI, *CI* confidence interval.

**Table 3:** Associations of maternal gestational hypertension with overweight/obesity in adolescent offspring (univariate and multivariate analyses).

Variables	Overweight/obesity		
	OR (95% CI)	aOR^1^ (95% CI)	aOR^2^ (95% CI)
Gestational hypertension			
No	1.00	1.00	1.00
Yes	1.50 (1.15–1.97)	1.51 (1.15–1.98)	1.41 (1.04–1.91)
	P = 0.003	P = 0.003	P = 0.029

*OR* crude odds ratio, *aOR*^*1*^ adjusted odds ratios for age and sex, *aOR*^*2*^ adjusted odds ratios for age, sex, birth weight, and maternal pre/early pregnancy BMI, *CI* confidence interval.

## Discussion

To our knowledge, this is the first study to examine the associations of maternal GH with high BP (prehypertension and hypertension) and overweight/obesity among schoolchildren aged 12–15 years not only in Lithuania but also in all the Baltic states. In the present study, the prevalence of GH was 7.7%, and thus it was higher than that observed in other published studies^33,37,39,40^, yet lower than previously reported^32,35,38^ and similar to that found by other scientists^42^. The results of our study are consistent with those of previous studies that demonstrated significant associations of maternal GH with advanced maternal age^43,44^, maternal obesity^44,45^, and cardiac diseases^46^. In our study, children born to women with GH had lower Apgar scores at 1 and 5 min and lower mean gestational age at birth, and in adolescence, they were heavier, more frequently had overweight/obesity, had a greater WHtR, and had higher BP levels than children of women without GH did. Our data is in line with the findings from other population studies where offspring of mothers with GH had greater mean BMI, WC, SBP, and DBP during childhood as compared to children of mothers who were without hypertensive disorders of pregnancy^32,35^. Fraser et al. found higher mean SBP and DBP as well as BMI in adolescent offspring of mothers with GH compared to those not exposed to hypertensive disorders of pregnancy in utero^36^. In the Western Australian Pregnancy Cohort (Raine) Study, mean SBP was significantly higher in the adolescent offspring of women with GH compared with controls^47^.

Univariate and multivariate logistic regression analyses of our data showed significant associations of maternal GH with offspring HBP (prehypertension and hypertension—either separately or combined) in adolescence. Our findings are also in line with those obtained in others previous studies that reported significant associations between maternal GH and offspring HBP. In the Project Viva cohort, maternal GH was related to higher offspring HBP in childhood^41^. The Avon Longitudinal Study of Parents and Children showed that maternal GH was associated with elevated BP in offspring at age 9–12 years, but no associations were observed with lipids, apolipoproteins, inflammatory markers, or endothelial dysfunction^32^. The Northern Finland Birth Cohort 1986 indicated that 16 year-old adolescents of mothers with GH had higher BP (SBP, DBP, and MAP) and tended to have higher cholesterol and apolipoprotein B values compared to the offspring of normotensive mothers^37^. The Born in Bradford cohort study found that GH was associated with higher SBP and DBP at age 4–5 in Pakistani children^42^. In the UK, a 9-year follow-up study revealed that women with GH had offspring with higher BP in childhood compared to those with no hypertensive disorders of pregnancy^35^. Staley et al. conducted a study in a large UK population birth cohort and reported an association between maternal GH and offspring BP at the age of 7 years^38^. However, other studies have reported different results. A population-based prospective cohort study from the Netherlands ascertained that maternal GH was associated with higher DBP, but not with SBP in school-aged children^40^. Rice et al. in a prospective observational follow-up study found that pregnancy-associated hypertension (GH and preeclampsia) in mothers who delivered at term was associated with a higher SBP in 5–10-year-old offspring but not with other cardiometabolic risk factors such as DBP, high-density lipoprotein cholesterol, triglycerides, glucose, homeostatic model assessment of insulin resistance, WC, or BMI^48^. The Tohoku Study of Child Development (Japan) reported no significant link between maternal GH and blood pressure in 7-year-old offspring^39^.

A meta-analysis of epidemiological studies has linked maternal hypertensive disorders with overweight and obesity in adult offspring^30^, but very little data are available on the association of maternal GH with overweight/obesity during childhood and adolescence. Our study also confirmed that maternal GH was associated with increased odds of overweight/obesity in the offspring during adolescence, and these findings are in agreement with the findings of other researchers. The results from a meta-analysis of prospective pregnancy/birth cohort studies including mothers and children from Europe and North America showed that GH was associated with higher odds of overweight and obesity in late childhood (from age 10.0 to 17.9 years) (aOR 1.49; 95% CI 1.30–1.70), compared to those born to mothers with an uncomplicated pregnancy. Even though additional adjustment for maternal pre- and early pregnancy BMI weakened the association, but it remained significant. The relationships were mainly explained by maternal pre-/early-pregnancy BMI^33^. In the Jiaxing Birth Cohort (JBC) study, increased maternal SBP and DBP during the second and the third trimesters of pregnancy among normotensive mothers were associated with an increased risk of overweight/obesity among 4–7-year-old offspring, but GH was not distinguished from preeclampsia^49^. However, other study found different results. According to the results of the Avon Longitudinal Study of Parents and Children, in the multivariate analysis, after adjustment for the age and sex of the offspring, GH was associated with an increased risk of offspring overweight/obesity; however, after additional adjustment for other potential confounders, including parental pre-pregnancy BMI, the positive association disappeared^35^.

Developmental programming of chronic diseases in late life such as cardiovascular, metabolic, and mental health disorders as well as obesity begins in utero and during early development^50^. Intrauterine growth restriction significantly increases the risk of cardiometabolic diseases in adulthood^51^. Physiological and biological explanations for the associations between hypertensive disorders during pregnancy and the offspring’s cardiovascular and metabolic health may include many aspects, such as endothelial dysfunction, increased oxidative stress^52^, changes in inflammatory markers during pregnancy^26,53^, maternal glucocorticoid metabolism, exogenous glucocorticoid exposure^26^, and intrauterine growth restriction^54^, causing changes in fetal adipose tissue morphology, metabolism, and hormone levels^55^. Those aspects also include a complex interplay of genetics, epigenetics, and environmental factors that may affect both the mother and the offspring^26^. Nevertheless, the mechanisms underlying these interactions remain poorly understood.

The strength of this study is that information on maternal GH and other maternal risk factors during pregnancy and infant anthropometric parameters was determined from medical records. This design of the study allowed us to minimize or avoid biases and errors, to reduce or prevent information bias, recall bias, and selection bias, and to provide reliable and accurate data and results. Another strength of this study is a sufficiently large and representative sample of adolescents. During the study, those with HBP ≥ 90th percentile on the first screening underwent a second screening.

However, our study has some limitations. We did not have any data on the children’s pubertal status, biochemical parameters, or maternal parity. Adolescents with overweight/obesity were combined into one group due to the small number of subjects with obesity. A very small number of children were born to mothers with preeclampsia. Differences in research methodologies, the number of visits and the number of BP measurements, sample size, the age of the examined schoolchildren, different definitions and criteria for overweight and obesity in children and adolescents, adjustment for different confounding variables in different studies, and demographic, socioeconomic, and geographic disparities between different populations make the comparison of the results complicated. Other researchers assessed the associations between maternal hypertensive disorders of pregnancy (including pre-eclampsia and GH), but not the subcategories of preeclampsia and GH separately or offspring HBP and overweight/obesity, which made a more accurate comparison of the results among studies difficult.

Our study results have an important impact on the development of strategies for public health and preventive medicine. The prevalence of HBP^8^, overweight, and obesity^11^ among children and adolescents has increased during the recent decades. Moreover, these childhood risk factors are associated with an increased risk of adverse cardiovascular events and mortality in adult life^56^. In order to develop HBP and overweight/obesity prevention and intervention strategies, it is essential to identify various risk factors including prenatal, demographic, lifestyle, and other factors that are associated with the development of cardiometabolic diseases in childhood, adolescence, and in later life.

The findings of our study suggest that it is very important for adolescent offspring born to mothers with GH to have their blood pressure and body weight checked regularly, even in the absence of other risk factors. Our findings expand the knowledge base on the effects of maternal risk factors during pregnancy on HBP and overweight/obesity in the offspring and are essential in the field of preventive medicine and public health when maintaining and prioritizing health and preventing cardiovascular and metabolic diseases. Moreover, a prevention program might focus more on maternal healthy lifestyle, healthy nutrition, physical activity, and the reduction of obesity before pregnancy in order to reduce or prevent adverse maternal and fetal outcomes.

## Conclusions

The findings of our study suggest that adolescent offspring of mothers with GH had significantly higher odds of HBP (prehypertension and hypertension, either separately or combined) and overweight/obesity, compared to those born to normotensive mothers.

## Methods

### Study population

For the present data analysis, we selected data of 5530 12–15-year-old subjects living in Kaunas city, the second largest city of Lithuania, who participated in the cross-sectional study of “Prevalence and Risk Factors of HBP in 12–15-Year-Old Lithuanian Children and Adolescents (Study 1)” which were carried out from November 2010 to April 2012. The methods and results of our study were described in previous publication^57–60^. Two-stage cluster sampling design was used to select the study sample of adolescents (grades 6–9; ages 12–15 years). In total, 56 schools of Kaunas city participated in the research project; in each school, classes from grades 6 to 9, and in each class, schoolchildren aged 12–15 years were selected.

Of the selected 5530 subjects, 97 were excluded from the final data analysis due to the presence of any of these conditions: endocrine diseases, kidney diseases, congenital heart defects, and cardiovascular diseases, and 16 more subjects were excluded due to missing anthropometric measurements. Additionally, subjects with missing data on birth weight and gestational age (N = 490), and multiple births (N = 108) were excluded. Thus, the final number of singleton subjects included in the current statistical analysis was 4819.

Kaunas Regional Ethics Committee for Biomedical Research at the Lithuanian University of Health Sciences approved the study (protocol No. BE–2–69). A written informed consent for participation was obtained from each subject’s parent or guardian. All methods were applied in accordance with relevant guidelines and regulations. Maternal and infant data were collected from medical records. Also, data on diseases and conditions were obtained from the subjects’ medical certificates (Form No.027-1/a). All BP and anthropometric measurements were performed at the participants’ schools by the same team of trained research staff (physicians, researchers, and research assistants).

### Blood pressure measurements

In the present study, BP was measured using an automatic BP monitor (OMRON M6; OMRON HEALTHCARE CO., LTD, Kyoto, Japan) with appropriately sized cuffs (17–22 cm; 22–32 cm; and 32–42 cm), three times with a 5-min rest interval between the measurements in the morning hours (8:30 am to 11:30 am) by a doctor who did not wear a white coat. The average of three BP measurements was calculated. BP measurements were performed with the subject being in a sitting position after the subjects had been sitting still for 10 min. Furthermore, in the morning of the examination day prior to the measurements, the schoolchildren were advised to avoid physical exercises and consuming caffeine and/or energy drinks. All subjects with HBP (≥ 90th percentile) during the first screening participated in the second screening of BP measurements conducted over a period of several weeks.

Definitions and classifications of BP levels were used as proposed by “The Fourth Report on the Diagnosis, Evaluation and Treatment of High Blood Pressure in Children and Adolescents”^61^. According to BP charts based on age, sex, and the height percentile, normal BP was defined as systolic blood pressure (SBP) and diastolic blood pressure (DBP) below the 90th percentile, while HBP was defined as average SBP or DBP levels ≥ 90th percentile. Prehypertension was defined as average SBP or DBP levels ≥ 90th percentile, but < 95th percentile, and hypertension was defined as mean SBP or DBP ≥ 95th percentile.

The mean arterial pressure (MAP) was calculated according to the following formula: MAP = (SBP + (2 × DBP))/3^62^. The pulse pressure (PP) was calculated as SBP minus DBP.

### Anthropometric measurements

Weight (to the nearest 0.1 kg) and body height (to the nearest 0.1 cm) were measured using a balance beam scale and a portable stadiometer (SECA measuring equipment) with the participants wearing no shoes and only light clothing. Waist circumference (to the nearest 0.5 cm) was measured at a level midway between the lower rib margin and the iliac crest using a flexible measuring tape. The body mass index (BMI) was calculated as the weight in kilograms divided by the height in meters squared (kg/m^2^). Normal weight, overweight, and obesity were defined using the International Obesity Task Force (IOTF) age- and sex-specific cut-off points^63^. The tri-ponderal mass index (TMI) was calculated as the weight in kilograms divided by the height in meters cubed (kg/m^3^)^64^. The waist-to-height ratio (WHtR) was calculated as the WC (cm) divided by height (cm).

### Maternal and infant health data

Mothers’ information on sociodemographic characteristics (age at delivery and education level), GH, heart diseases, diabetes mellitus, and pre/early pregnancy body weight status was obtained from medical records. Women were divided into groups by age at delivery (less than 20 years, 20–34 years, and 35 years or older), education level (primary/secondary or advanced vocational or higher). Gestational hypertension was defined as elevated systolic blood pressure ≥ 140 mmHg and/or diastolic blood pressure ≥ 90 mmHg on at least two occasions four hours apart, developing after 20 weeks of gestation with no signs of proteinuria and without evidence of end-organ damage in a previously normotensive woman. Maternal heart diseases included the following diseases and disorders: congenital heart defects, acquired heart defects, cardiomyopathies, arrhythmias, endocarditis and pericarditis, and ischemic heart disease. According to pre-pregnancy and early pregnancy (less than 12 weeks) body weight status, mothers were grouped into 2 groups: those with and without obesity. Maternal obesity was defined as a BMI ≥ 30.

Study subjects were born in Kaunas city during the years 1995 to 1998. Infant data (sex, birth date, birth weight, gestational age, birth length, and Apgar scores at 1 min and 5 min) were extracted from medical records. Newborns were categorized into three groups based on Lithuanian national birthweight percentiles by gestational age and sex: small for gestational age (SGA: BW < 10th percentile), appropriate for gestational age (AGA: BW ≥ 10th– ≤ 90th percentile), and large for gestational age (LGA: BW > 90th percentile)^65^. Normal birth weight was defined as a birth weight between ≥ 2500 g and ≤ 4000 g, while high BW was defined as birth weight > 4000 g^66^. The weight/length ratio of the newborns was calculated as weight (kg) divided by length (m)^67^. The ponderal index (PI) was calculated by applying the following formula: PI = birth weight (g) × 100/birth length^3^ (cm^3^).

### Statistical analysis

Categorical variables were presented as numbers (n) and percentages (%) and were compared using the chi-square (χ^2^) test and the z test with Bonferroni corrections. The normality of the distribution of the continuous variables was tested by applying the Kolmogorov–Smirnov test. Means and standard deviations (SD) were presented for the normally distributed continuous variables. The t-test was used to compare the mean values of normally distributed variables.

Univariate and multivariate logistic regression analyses were conducted for both sexes combined to evaluate the associations of maternal GH with HBP and overweight/obesity in offspring during adolescence. Crude odds ratios (OR) and adjusted odds ratios (aOR) and their 95*%* confidence intervals (CI) were calculated. In the multivariate analysis, models were designed for the evaluation of the associations of maternal GH with high BP: in the first model, ORs were adjusted for age and sex, and in the second model, ORs were adjusted for age, sex, birth weight, adolescent BMI, and maternal pre/early pregnancy BMI. Models were also created for the evaluation of the associations of maternal GH with overweight/obesity: in the first model, ORs were adjusted for age and sex, and in the second model, ORs were adjusted for age, sex, birth weight, and maternal pre/early pregnancy BMI.

Statistical data analysis was performed using the statistical software package SPSS version 20 for Windows. P values of less than 0.05 were regarded as statistically significant.

## Supplementary Information

Supplementary Information.

## Data Availability

According to the Statute of the Lithuanian University of Health Sciences, the authors cannot share the data underlying this study. For inquires on the data, researchers should first contact the owner of the database, the Lithuanian University of Health Sciences.
