# Supplementary material for: Associations of maternal gestational hypertension with high blood pressure and overweight/obesity in their adolescent offspring: a retrospective cohort study
Source: Sci Rep. 2022 Mar 8;12:3800. doi: 10.1038/s41598-022-07903-z (PMC8904808; doi:10.1038/s41598-022-07903-z)
Supplement: Supplementary file 1 — Supplementary Information. [file 41598_2022_7903_MOESM1_ESM.docx]

**Associations of maternal gestational hypertension with high blood pressure and overweight/obesity in their adolescent offspring: a retrospective cohort study**

Renata Kuciene, Virginija Dulskiene

**Supplementary Table 1. Maternal and offspring characteristics according to offspring blood pressure levels in adolescence**

| **Variables** | **NBP (n=3,581)** | **HBP (n=1,238)** | ***p**** |
| --- | --- | --- | --- |
| ***Maternal characteristics*** |  |  |  |
| Maternal age at delivery (years), n (%): |  |  |  |
| <20 | 275 (7.7) | 90 (7.3) | 0.575 |
| 20–34 | 2,996 (83.6) | 1,051 (84.9) |  |
| ≥35 | 310 (8.7) | 97 (7.8) |  |
| Maternal age at delivery (years), mean ± SD | 26.88±5.33 | 26.84±5.16 | 0.983 |
| Maternal education: |  |  |  |
| Primary/ Secondary | 1,859 (52.1) | 653 (53.0) | 0.579 |
| Advanced vocational/ Higher | 1,710 (47.9) | 579 (47.0) |  |
| Maternal gestational  hypertension |  |  |  |
| No | 3,347 (93.5) | 1,101 (88.9)^*^ | **<0.001** |
| Yes | 234 (6.5) | 137 (11.1)^*^ |  |
| Maternal diabetes mellitus |  |  |  |
| No | 3,559 (99.4) | 1,217 (98.3)^*^ | **<0.001** |
| Yes | 22 (0.6) | 21 (1.7)^*^ |  |
| Maternal body weight status, n (%): |  |  |  |
| No obesity | 3,327 (92.9) | 1,114 (90.0)^*^ | **0.001** |
| Obesity | 254 (7.1) | 124 (10.0)^*^ |  |
| Maternal heart diseases, n (%): |  |  |  |
| No | 3,210 (89.6) | 1,110 (89.7) | 0.983 |
| Yes | 371 (10.4) | 128 (10.3) |  |
| ***Neonates’ characteristics*** |  |  |  |
| Sex, n (%): |  |  |  |
| Boys | 1,458 (40.7) | 749 (60.5)^*^ | **<0.001** |
| Girls | 2,123 (59.3) | 489 (39.5)^*^ |  |
| Birth weight categories (g), n (%): |  |  |  |
| ≤4,000 | 3,157 (88.2) | 1,021 (82.5)^*^ | **<0.001** |
| >4,000 | 424 (11.8) | 217 (17.5)^*^ |  |
| Birth weight for gestational age: |  |  |  |
| SGA | 321 (9.0) | 97 (7.8) | **<0.001** |
| AGA | 2,922 (81.6) | 972 (78.5)^*^ |  |
| LGA | 338 (9.4) | 169 (13.7)^*^ |  |
| Birth weight (g), mean ± SD | 3,479.53±507.44 | 3,542.21±518.79 | **0.002** |
| Gestational age (weeks), mean ± SD | 39.52±1.50 | 39.39±1.58 | **0.004** |
| Birth length (cm), mean ± SD | 50.91±2.33 | 51.13±2.18 | **0.003** |
| Weight/length ratio, (kg/m), mean ± SD | 6.81±0.83 | 6.91±0.83 | **0.006** |
| BMI, (kg/m^2^), mean ± SD | 13.36±1.42 | 13.49±1.37 | 0.053 |
| PI, (g/cm^3^), mean ± SD | 2.62±0.27 | 2.64±0.26 | 0.330 |
| Apgar scores, mean (SD): |  |  |  |
| 1-minute Apgar score, mean ± SD | 8.42 ±1.12 | 8.41±0.98 | 0.085 |
| 5-minute Apgar score, mean ± SD | 9.10±0.99 | 9.11±0.82 | 0.165 |
| ***Adolescents’ characteristics:*** |  |  |  |
| Age (years), n (%): |  |  |  |
| 12–13 | 1,758 (49.1) | 478 (38.6)^*^ | **<0.001** |
| 14–15 | 1,823 (50.9) | 760 (61.4)^*^ |  |
| BMI categories, n (%): |  |  |  |
| Normal weight | 3,232 (90.3) | 890 (71.9)^*^ | **<0.001** |
| Overweight/obesity | 349 (9.7) | 348 (28.1)^*^ |  |
| Age (years), mean ± SD | 13.54±1.05 | 13.77±1.02 | **<0.001** |
| Weight (kg), mean ± SD | 51.91±10.57 | 61.71±20.60 | **<0.001** |
| Height (cm), mean ± SD | 164.39±9.23 | 169.22±9.61 | **<0.001** |
| BMI (kg/m^2^), mean ± SD | 19.08±2.82 | 21.53±9.25 | **<0.001** |
| TMI (kg/m^3^), mean ± SD | 11.63±1.76 | 12.78±6.55 | **<0.001** |
| WC (cm), mean ± SD | 65.88±6.78 | 71.14±8.82 | **<0.001** |
| WHtR, mean ± SD | 0.401±0.04 | 0.421±0.05 | **<0.001** |
| SBP (mm Hg), mean ± SD | 112.49±9.07 | 137.96±10.60 | **<0.001** |
| DBP (mm Hg), mean ± SD | 63.98±6.56 | 71.75±8.20 | **<0.001** |
| MAP (mm Hg), mean ± SD | 80.15±6.31 | 93.82±7.21 | **<0.001** |
| PP (mm Hg), mean ± SD | 47.80±7.56 | 65.95±11.74 | **<0.001** |

NBP – normal blood pressure; HBP – high blood pressure; SGA – small for gestational age; AGA – appropriate for gestational age; LGA – large for gestational age; BMI – body mass index, PI – ponderal index; WC – waist circumference, TMI – tri-ponderal mass index; WHtR – waist-to-height ratio, SBP – systolic blood pressure, DBP – diastolic blood pressure, MAP – mean arterial pressure, PP – pulse pressure.

Values are numbers (percentages) and mean ± SD (standard deviation).

* P <0.05 vs. the normotensive group (z test).

**Supplementary Table 2. Maternal and offspring characteristics according to the BMI categories of the offspring in adolescence**

| **Variables** | **Normal weight (n=4,122)** | **Overweight/obesity (n=697)** | ***p**** |
| --- | --- | --- | --- |
| ***Maternal characteristics*** |  |  |  |
| Maternal age at delivery (years), n (%): |  |  |  |
| <20 | 316 (7.7) | 49 (7.0) | 0.752 |
| 20–34 | 3,455 (83.8) | 592 (85.0)^*^ |  |
| ≥35 | 351 (8.5) | 56 (8.0) |  |
| Maternal age at delivery (years), mean ± SD | 26.86±5.28 | 26.95±5.30 | 0.663 |
| Maternal education: |  |  |  |
| Primary/ Secondary | 2,143 (52.2) | 369 (53.0) | 0.691 |
| Advanced vocational/ Higher | 1,962 (47.8) | 327 (47.0) |  |
| Maternal gestational  hypertension |  |  |  |
| No | 3,824 (92.8) | 624 (89.5)^*^ | **0.003** |
| Yes | 298 (7.2) | 73 (10.5)^*^ |  |
| Maternal diabetes mellitus |  |  |  |
| No | 4,089 (99.2) | 687 (98.6) | 0.100 |
| Yes | 33 (0.8) | 10 (1.4) |  |
| Maternal body weight status, n (%): |  |  |  |
| No obesity | 3,861 (93.7) | 580 (83.2)^*^ | **<0.001** |
| Obesity | 261 (6.3) | 117 (16.8)^*^ |  |
| Maternal heart diseases, n (%): |  |  |  |
| No | 3,691 (89.5) | 629 (90.2) | 0.575 |
| Yes | 431 (10.5) | 68 (9.8) |  |
| ***Neonates’ characteristics*** |  |  |  |
| Sex, n (%): |  |  |  |
| Boys | 1,826 (44.3) | 381 (54.7)^*^ | **<0.001** |
| Girls | 2,296 (55.7) | 316 (45.3)^*^ |  |
| Birth weight categories (g), n (%): |  |  |  |
| ≤4,000 | 3,614 (87.7) | 564 (80.9)^*^ | **<0.001** |
| >4,000 | 508 (12.3) | 133 (19.1)^*^ |  |
| Birth weight for gestational age: |  |  |  |
| SGA | 376 (9.1) | 42 (6.0)^*^ | **<0.001** |
| AGA | 3,345 (81.2) | 549 (78.8) |  |
| LGA | 401 (9.7) | 106 (15.2)^*^ |  |
| Birth weight (g), mean ± SD | 3,477.05±507.67 | 3,605.54±517.58 | **<0.001** |
| Gestational age (weeks), mean ± SD | 39.48±1.53 | 39.53±1.51 | 0.548 |
| Birth length (cm), mean ± SD | 50.91±2.32 | 51.28±2.10 | **<0.001** |
| Weight/length ratio, (kg/m), mean ± SD | 6.81±0.83 | 7.01±0.84 | **<0.001** |
| BMI, (kg/m^2^), mean ± SD | 13.35±1.40 | 13.65±1.41 | **<0.001** |
| PI, (g/cm^3^), mean ± SD | 2.62±0.27 | 2.66±0.27 | **<0.001** |
| Apgar scores, mean (SD): |  |  |  |
| 1-minute Apgar score, mean ± SD | 8.42 ±1.11 | 8.37±0.93 | **0.003** |
| 5-minute Apgar score, mean ± SD | 9.11±0.98 | 9.08±0.76 | **0.015** |
| ***Adolescents’ characteristics:*** |  |  |  |
| Age (years), n (%): |  |  |  |
| 12–13 | 1,874 (45.5) | 362 (51.9)^*^ | **0.002** |
| 14–15 | 2,248 (54.5) | 335 (48.1)^*^ |  |
| BP categories, n (%): |  |  |  |
| NBP | 3,232 (78.4) | 349 (50.1)^*^ | **<0.001** |
| Prehypertension | 363 (8.8) | 130 (18.7)^*^ |  |
| Hypertension | 527 (12.8) | 218 (31.2)^*^ |  |
| BP categories, n (%): |  |  |  |
| NBP | 3,232 (78.4) | 349 (50.1)^*^ | **<0.001** |
| HBP | 890 (21.6) | 348 (49.9)^*^ |  |
| Age (years), mean ± SD | 13.62±1.04 | 13.48±1.04 | **0.001** |
| Weight (kg), mean ± SD | 51.54±9.27 | 71.54±24.58 | **<0.001** |
| Height (cm), mean ± SD | 165.47±9.52 | 166.60±9.77 | **0.005** |
| BMI (kg/m^2^), mean ± SD | 18.69±2.05 | 25.74±11.55 | **<0.001** |
| TMI (kg/m^3^), mean ± SD | 11.31±1.21 | 15.57±8.35 | **<0.001** |
| WC (cm), mean ± SD | 65.35±5.68 | 78.36±8.68 | **<0.001** |
| WHtR, mean ± SD | 0.395±0.03 | 0.471±0.05 | **<0.001** |
| SBP (mm Hg), mean ± SD | 117.63±14.12 | 127.33±14.83 | **<0.001** |
| DBP (mm Hg), mean ± SD | 65.55±7.64 | 68.46±8.21 | **<0.001** |
| MAP (mm Hg), mean ± SD | 82.91±8.62 | 88.08±8.99 | **<0.001** |
| PP (mm Hg), mean ± SD | 51.51±11.40 | 58.12±12.93 | **<0.001** |

NBP – normal blood pressure; HBP – high blood pressure; SGA – small for gestational age; AGA – appropriate for gestational age; LGA – large for gestational age; BMI – body mass index, PI – ponderal index; WC – waist circumference, TMI – tri-ponderal mass index; WHtR – waist-to-height ratio, SBP – systolic blood pressure, DBP – diastolic blood pressure, MAP – mean arterial pressure, PP – pulse pressure.

Values are numbers (percentages) and mean ± SD (standard deviation).

* P <0.05 vs. the normal weight group (z test).
